# The long-term cardiovascular safety and efficacy of glucagon-like peptide-1 (GLP-1) receptor agonists in high-risk cardiovascular populations: a systematic review and meta-analysis

**DOI:** 10.1186/s40842-026-00295-3

**Published:** 2026-05-01

**Authors:** Kezia Peter, Ocin Roka, Emma Sepp, Maya Warburton, Jufen Zhang, Simon C. Cork

**Affiliations:** School of Medicine, Anglia Ruskin University, Bishop Hall Road, Chelmsford, CM1 1SQ UK

**Keywords:** GLP-1 receptor agonists, Major adverse cardiovascular events (MACE), Cardiovascular outcome trials (CVOTS), Type 2 diabetes, Meta-analysis, Cardioprotection

## Abstract

**Background:**

Glucagon-like peptide-1 receptor agonists (GLP-1RAs) are widely used for the management of type 2 diabetes and obesity, yet their long-term cardiovascular effects in high-risk populations continue to be actively evaluated. Given emerging evidence of both metabolic and direct cardiovascular actions, a comprehensive synthesis of cardiovascular outcome trial data is required to clarify the efficacy and safety of this drug class.

**Methods:**

We conducted a systematic review and meta-analysis of randomised, placebo-controlled cardiovascular outcome trials evaluating GLP-1RAs in adults at high cardiovascular risk. Searches of PubMed, Embase (via OVID), and the Cochrane Library were performed for studies published between January 2015 and May 2025, in accordance with PRISMA 2020 guidelines. Eligible trials included ≥ 3,000 participants with a minimum follow-up of 12 months. The primary outcome was major adverse cardiovascular events (MACE). Secondary outcomes included cardiovascular mortality, all-cause mortality, non-fatal myocardial infarction, non-fatal stroke, hospitalisation for heart failure, and adverse events. Hazard ratios (HRs) with 95% confidence intervals (CIs) were pooled using random-effects meta-analysis. Risk of bias was assessed using the Cochrane RoB 2 tool, and certainty of evidence was evaluated using GRADE.

**Results:**

Eleven cardiovascular outcome trials comprising 91,490 participants were included, with a mean follow-up of 2.7 years. GLP-1RA treatment was associated with a significant reduction in MACE compared with placebo (HR 0.86, 95% CI 0.81–0.92). Meta-analysis also demonstrated significant reductions in cardiovascular mortality, all-cause mortality, non-fatal myocardial infarction, non-fatal stroke, and hospitalisation for heart failure. GLP-1RAs did not materially increase the risk of severe hypoglycaemia or acute pancreatitis, while gastrointestinal adverse effects were consistently more frequent.

**Conclusions:**

GLP-1 receptor agonists significantly reduce major cardiovascular events and mortality in high-risk populations, with a favourable long-term safety profile. These findings support the broader integration of GLP-1RAs into cardiovascular risk reduction strategies beyond glycaemic control.

**Graphical Abstract:**

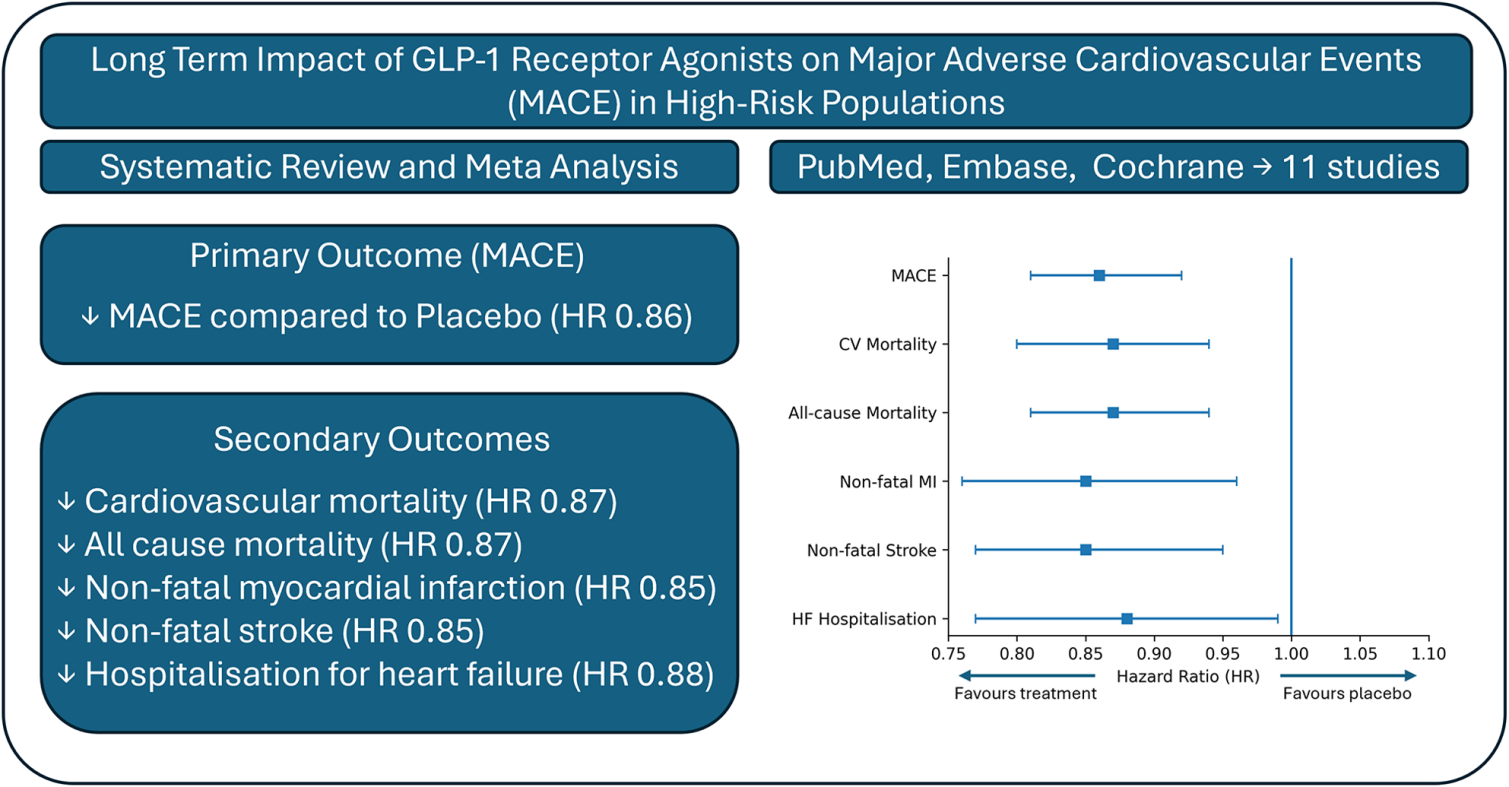

**Supplementary Information:**

The online version contains supplementary material available at 10.1186/s40842-026-00295-3.

## Introduction

Cardiovascular diseases (CVDs) remain the leading cause of mortality worldwide, accounting for approximately 17.9 million deaths annually, representing around 32% of all global deaths [1]. Despite advances in prevention and treatment, the burden of CVD continues to rise, particularly among high-risk populations [2]. The UK’s National Institute for Health and Care Excellence (NICE) defines high-risk individuals as those with a 10-year risk of a first cardiovascular event of 10% or above, often calculated using tools such as QRISK3 [3].

Glucagon-like peptide-1 receptor agonists (GLP-1RAs) were initially developed for the treatment of type 2 diabetes mellitus and now have a key role in obesity management. This class of medication has emerged as having potential cardiovascular benefits. While their abilities to improve glycaemic control and promote weight loss are well established, increasing attention has focused on their potential to directly influence cardiovascular outcomes.

Several mechanisms have been proposed to explain these newfound cardioprotective effects of GLP-1RAs. These therapeutic agents contribute to improvements in key cardiovascular risk factors, including reductions in body weight, blood pressure, and glycaemic variability, all of which are independently associated with decreased cardiovascular risk [4, 5]. Beyond these metabolic effects, GLP-1RAs may exert direct actions on the cardiovascular system, including improved endothelial function, reduced inflammation, and modulation of atherosclerotic processes [4]. These effects provide a possible explanation for their role in reducing cardiovascular events.

Importantly, these mechanistic hypotheses are supported by evidence from large, randomised controlled trials, which has demonstrated that GLP-1RAs reduced major adverse cardiovascular events (MACE), including myocardial infarction, stroke and cardiovascular death, particularly in high-risk individuals such as those with type 2 diabetes mellitus. This combination of mechanistic plausibility and clinical evidence has demonstrated the potential for GLP-1RAs as a possible therapeutic strategy for improving cardiovascular prognosis.

This systematic review and meta-analysis aims to investigate whether GLP-1RAs provide long-term cardiovascular protective benefits in patients at high risk of major cardiovascular events.

## Methodology

### Study design

This study is a systematic review and meta-analysis of cardiovascular outcome trials (CVOTs) evaluating the long-term safety and efficacy of glucagon-like peptide-1 receptor agonists (GLP-1 RAs) in populations at high cardiovascular risk. The protocol (Supplementary Appendix A) was developed in accordance with Preferred Reporting Items for Systematic Reviews and Meta-Analyses (PRISMA) 2020 guidelines [6] and has been published on PROSPERO (ID: CRD420251051447).

### Literature search

A comprehensive literature search was conducted in May 2025 across PubMed, Embase via OVID and the Cochrane Library from 01 January 2015 to 12 May 2025. Search terms included a combination of Medical Subject Headings (MeSH) and free-text terms, such as ‘GLP-1 receptor agonist’, ‘semaglutide’, ‘liraglutide’, ‘dulaglutide’, ‘cardiovascular outcomes’ and ‘major adverse cardiovascular events. The complete search strategy can be found in Supplementary Appendix B.

To streamline the review process, Covidence [7], an automation tool for systematic reviews, was used to manage reference screening and selection. All retrieved citations were imported into Covidence, where duplicates were automatically removed. Additional duplicates were identified and removed manually using RefWorks.

### Eligibility criteria

Studies were included if they met the following criteria, according to the PICOS framework:

#### Population

Adults (≥ 18 years) only at high risk for cardiovascular disease (CVD) or with established CVD (coronary artery disease, heart failure, prior myocardial infarction, stroke).

Participants with comorbid conditions associated with cardiovascular risk, including type 2 diabetes, pre-diabetes, obesity, hypertension and dyslipidaemia. Individuals without diabetes but with multiple cardiovascular risk factors were also included where defined as high cardiovascular risk in the original trials. Cardiovascular risk classification was based on the eligibility criteria used in each cardiovascular outcome trial, rather than a single risk prediction tool such as the Framingham Risk Score. These criteria included established atherosclerotic cardiovascular disease, recent acute coronary syndrome or the presence of risk factors and target-organ damage e.g. microalbuminuria, hypertension with left ventricular hypertrophy, reduced eGFR for kidney function or abnormal ankle-brachial index.

#### Intervention

Studies evaluating GLP-1 receptor agonists (Liraglutide, Lixisenatide, Semaglutide, Exenatide, Albiglutide, Dulaglutide, Efpeglenatide).

#### Comparator

Placebo.

#### Outcome

Primary: Cardiovascular outcomes - major adverse cardiovascular events [MACE].

Secondary: cardiovascular mortality, non-fatal myocardial infarction, non-fatal stroke, heart failure.

Adverse Effects: Safety outcomes (severe hypoglycaemia, pancreatitis, GI side effects).

#### Study design

Randomised controlled trial (RCT).

Minimum total sample size of 3000 participants.

Minimum follow-up period of 12 months to ensure adequate evaluation of long-term cardiovascular outcomes.

#### Other

Peer-reviewed articles, published in English.

Studies published within the last 10 years to reflect current clinical practices and drug development.

### Study selection

**Fig. 1:**
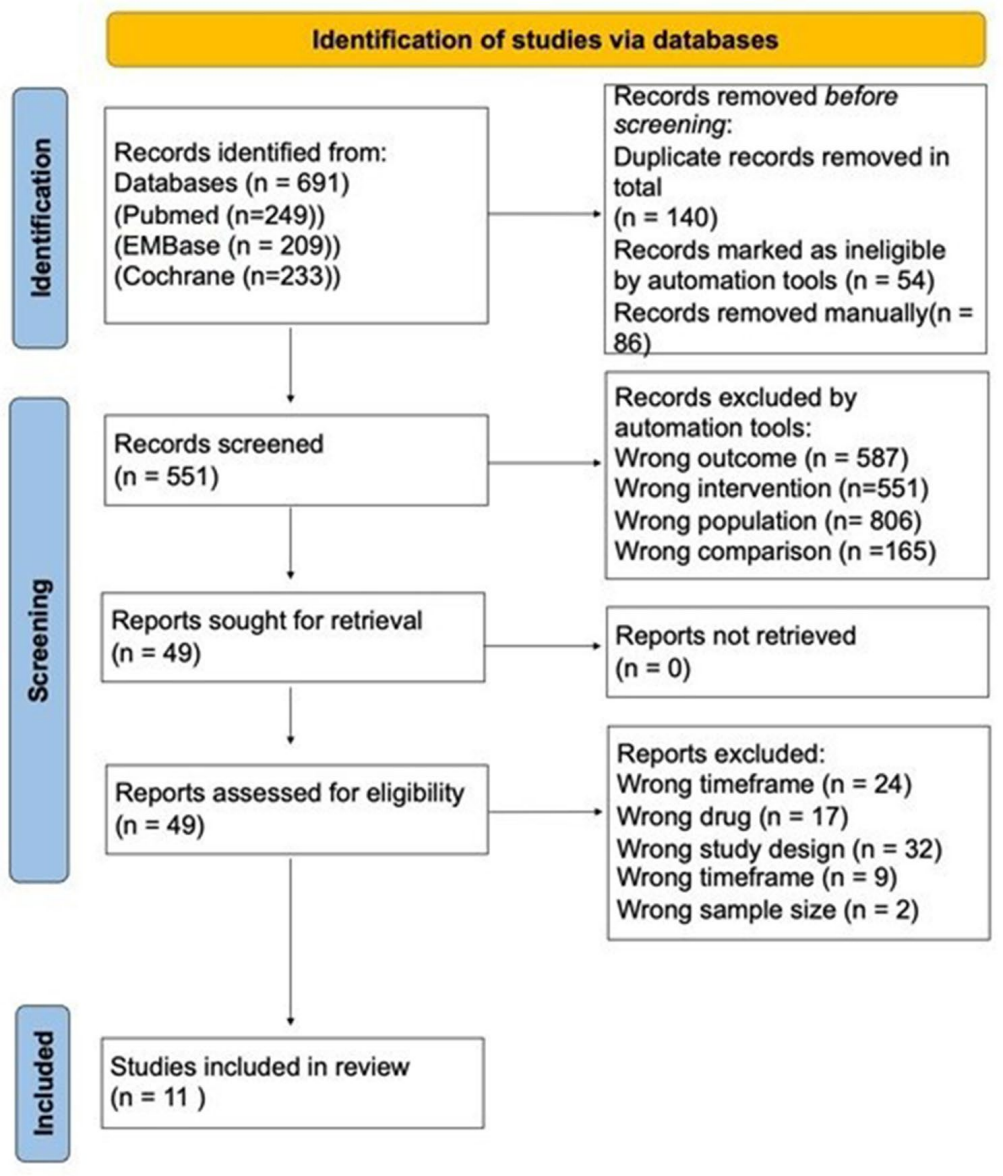
PRISMA (Preferred reporting items for systematic reviews and meta-analyses) 2020 flowchart illustrating literature searching methods [6]

Two reviewers (KP and OR) independently screened all titles and abstracts, followed by full-text assessment of potentially eligible studies. Conflicts were resolved through discussion and referral to third person (SCC) as necessary. The study selection process is illustrated using PRISMA 2020 flow diagram (Fig. 1).

### Data extraction and synthesis

A detailed data extraction table was developed on Microsoft Excel and data on key study characteristics and outcomes were collected. For each included study, data was extracted on study design, baseline population characteristics, intervention details (type and dose of GLP-1 receptor agonists), comparator, mean follow-up duration and reported cardiovascular outcomes. This included MACE, cardiovascular mortality, all-cause mortality, myocardial infarction, stroke, hospitalisation for heart failure and adverse effects, including acute pancreatitis, severe hypoglycaemia and gastrointestinal side-effects (Table 1).

Two reviewers (KP and OR) independently extracted the data. Discrepancies were resolved through discussion and referral to third person (SCC) as necessary. Results were grouped according to primary and secondary outcomes. A narrative synthesis was conducted to summarise findings across studies. Statistical analysis was performed using STATA statistical software (version 18, StataCorp LLC). Hazard ratios (HRs) with 95% confidence intervals (CIs) were used to assess primary (MACE) and secondary outcomes (all-cause mortality, CV death, non-fatal MI, non-fatal stroke, and HF hospitalisation) between the two treatment groups. The HRs and 95% CIs were directly extracted for each outcome from the studies. The random-effects model (DerSimonian-Laird method) was applied across studies. I-squared measure was used to quantify the heterogeneity, with I^2^ > 50% indicating substantial heterogeneity. Forest plots are used to represent the results generated from the random-effects meta-analysis graphically. The pooled HR and the degree of heterogeneity are presented. To reduce the potential for heterogeneity due to different follow-up lengths among the trials, meta-regression analysis was conducted.

In addition, post-hoc sensitivity analyses were performed by excluding the prediabetic population (SELECT trial [8]) in Fig. 6. A post-hoc subgroup analysis was conducted to investigate trials evaluating semaglutide separately to assess whether individual interventions contributed disproportionately to the overall findings (Supplementary Appendix D). An additional post-hoc sensitivity analysis was performed by excluding the ITCA 650 trial [9] to investigate both primary and secondary outcomes to validate the robustness of the pooled analysis (Supplementary Appendix D). A p-value lower than 0.05 was considered as statistically significant. Publication bias was minimised by comprehensive literature searching.

### Risk of bias and quality assessment

The quality of each included study and outcomes was independently assessed by reviewers (KP and OR). Discrepancies were resolved through discussion and referral to third person (SCC) as necessary. The Cochrane Risk of Bias 2 (RoB2) tool [10] was used to evaluate bias across five domains: randomisation process, deviations from the intended interventions, missing outcome data, measurement of the outcome and selection of the reported result. Each study was assigned a risk of bias rating of ‘low risk’, ‘some concerns’ or ‘high risk’ in each domain and an overall rating.

The overall rating for risk of bias obtained through the RoB2 tool was included as part of the Grading of Recommendations, Assessment, Development and Evaluation (GRADE) approach [11]. The GRADE approach assessed the quality of the evidence for each outcome across five domains: risk of bias, inconsistency, indirectness, imprecision and publication bias. Each outcome was rated as very low, low, moderate and high certainty. A detailed summary of findings table was constructed to present the quality of evidence across the included outcomes and studies (Supplementary Appendix C).

## Results

### Study selection

A total of 645 studies were identified through database searches in PubMed, Ovid and The Cochrane Library. After removing duplicates and screening the titles and abstracts of these studies, 49 full-text studies were assessed for eligibility. Following this, 11 studies met the inclusion criteria and data was extracted for the systematic review. A PRISMA flow diagram [6] illustrating the selection process can be found in Fig. 1.

### Study characteristics

The included studies were published between 2016 and 2025, with sample sizes ranging from 3,183 to 17,604 participants. Across the 11 included studies, 91,490 participants were analysed. The pooled mean age was 63.8 (± 1.8) years and 66.1% of participants were male. Baseline characteristics of the included studies are summarised in Table 1. The median follow-up was 2.7 years, where the minimum median duration was 1.3 years, and the maximum was 5.4 years. All 11 studies were randomised placebo-controlled trials investigating GLP-1 receptor agonists in adults with high cardiovascular risk. Drugs investigated across these studies include: Liraglutide (*n* = 1, LEADER [12]); Lixisenatide (*n* = 1, ELIXA [13]); Semaglutide (*n* = 4, SUSTAIN-6 [14], PIONEER-6 [15], SELECT [8], SOUL [16]); Exenatide (*n* = 2, EXSCEL [17]), ITCA 650 [9]; Albiglutide (*n* = 1, HARMONY Outcomes [18]); Dulaglutide (*n* = 1, REWIND [19]); Efpeglenatide (*n* = 1, AMPLITUDE-O [20]).

The COX proportional hazards model was used by all 11 studies to determine the effect of the intervention on the outcome and to estimate hazard ratios and 95% confidence intervals. All studies are cardiovascular outcome trials (CVOTs) and reported on MACE as their primary outcomes. The studies reported on secondary outcomes, including all-cause mortality, fatal and non-fatal myocardial infarction (MI) and stroke, as well as hospitalisation for heart failure. Adverse events were also reported, including acute pancreatitis, severe hypoglycaemia and gastrointestinal side effects. A detailed summary of study characteristics is presented in a data extraction table (Table 1).

### Primary outcome

**Fig. 2:**
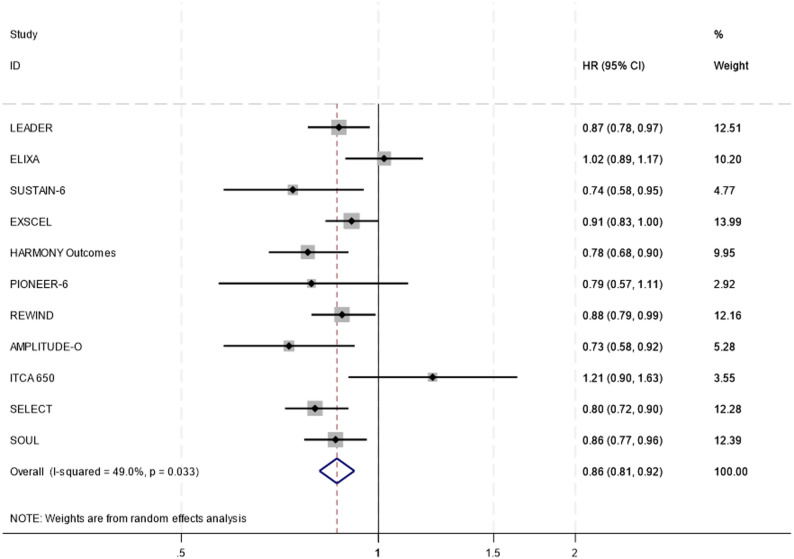
Forest plot diagram between GLP-1 receptor agonists and placebo for MACE events according to random effects analysis

The primary outcome, major adverse cardiovascular events (MACE), was reported in all 11 included studies, comprising of 91,490 participants with 4386 MACE events in the intervention group and 4884 MACE events in the placebo group. The mean duration of follow-up in the studies was 2.7 years. In the meta-analysis, GLP-1 receptor agonists reported a significant reduction in MACE compared with placebo (HR (95%CI): 0.86 (0.81–0.92); I² = 49%, *p* = 0.033) (Fig. 2).

In a sensitivity analysis excluding the SELECT trial, which was the only study enrolling pre-diabetic participants, the reduction in MACE remained consistent (HR (95% CI): 0.87(0.82–0.93); I² = 47.8%, *p* = 0.045), confirming that the cardioprotective effect was robust in participants with diabetes (Fig. 4). A subgroup analysis of the four trials investigating semaglutide as the intervention reported greater reduction in MACE than in the pooled analysis (HR (95% CI): 0.82(0.76–0.88)); I² = 0.0%, *p* = 0.656) (Supplementary Figure D5). A sensitivity analysis excluding the ITCA trial reported a similar reduction in MACE as the pooled analysis (HR (95%CI): 0.86 (0.81–0.90); I² = 38.7%, *p* = 0.100) (S).

### Secondary outcomes

**Fig. 3:**
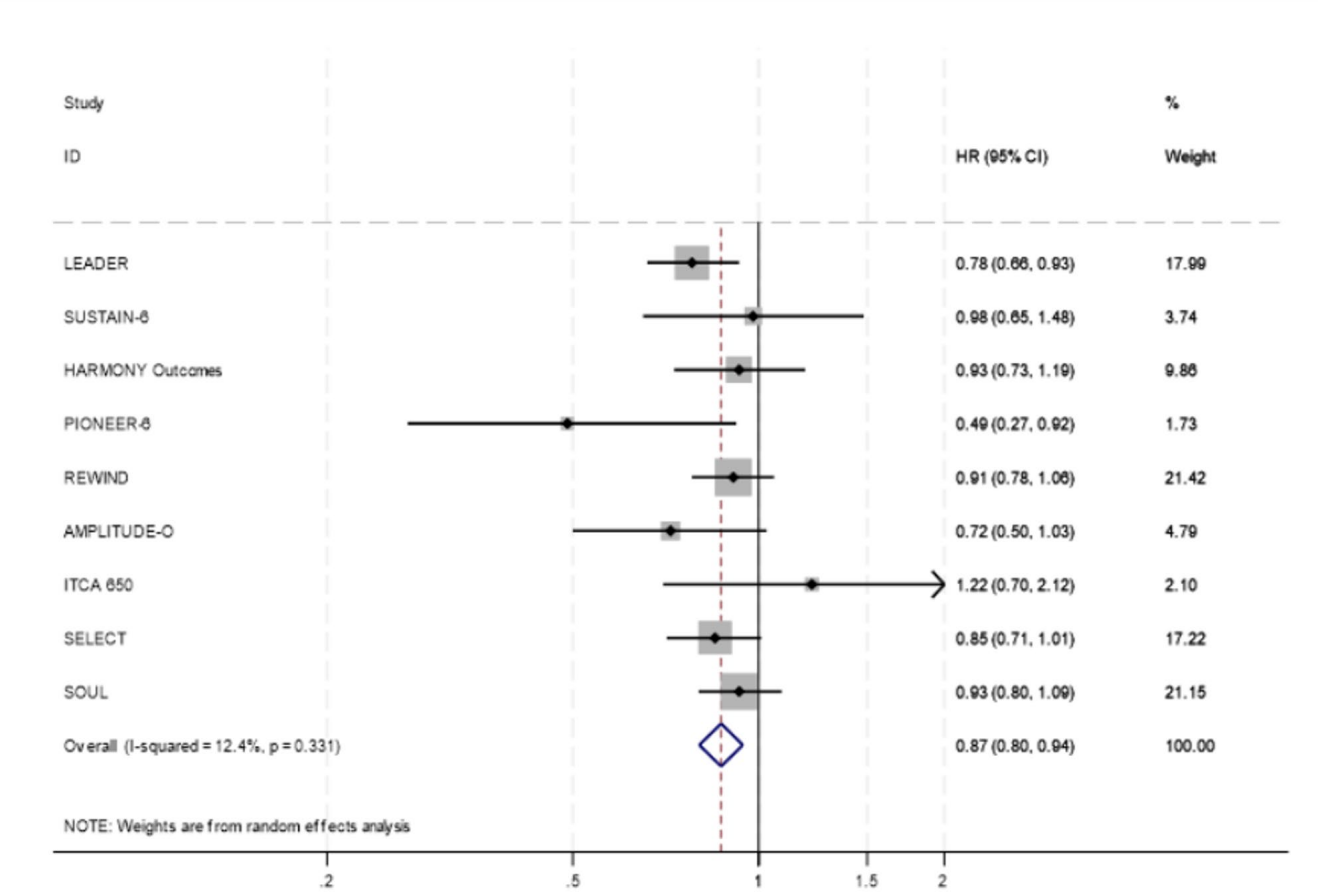
Forest plot diagram between GLP-1 receptor agonists and placebo for cardiovascular death according to random effects analysis

**Fig. 4:**
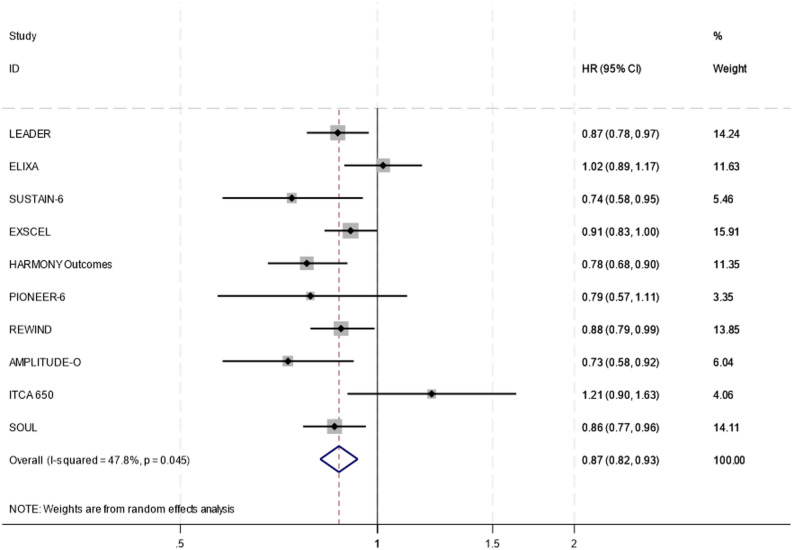
Forest plot diagram of sensitivity analyses performed by excluding the prediabetic population (SELECT trial)

Secondary outcomes included cardiovascular mortality, all-cause mortality, non-fatal myocardial infarction (MI) and stroke, as well as hospitalisation for heart failure. Detailed forest plots for all secondary outcomes, subgroup analysis and ITCA 650 sensitivity analysis are included in Supplementary Appendix D, except pooled analysis of cardiovascular mortality where this is included in Fig. 3 and SELECT trial sensitivity analysis where this is included in Fig. 4.

- Cardiovascular mortality was reported in 9 of the 11 studies. The pooled analysis demonstrated a significant reduction in cardiovascular mortality (HR (95% CI): 0.87 (0.80–0.94); I² = 12.4%, *p* = 0.331). A sensitivity analysis excluding the ITCA trial yielded consistent results (HR (95%CI): 0.86 (0.80–0.94); I² = 8.9%, *p* = 0.361).
- All-cause mortality was reported in 8 of the 11 studies. The pooled estimate showed a significant reduction in all-cause mortality (HR (95% CI): 0.87 (0.81–0.94); I² = 29.4%, *p* = 0.193). A sensitivity analysis excluding the ITCA trial yielded consistent results (HR (95%CI): 0.86 (0.81–0.93); I² = 21.2%, *p* = 0.267).
- Non-fatal MI was reported in 8 of the 11 studies with the pooled analysis demonstrating a modest reduction in risk (HR (95% CI): 0.85 (0.76–0.96); I² = 41.4%, *p* = 0.103). A sensitivity analysis excluding the ITCA trial yielded consistent results (HR (95%CI): 0.83 (0.75–0.93); I² = 29.0%, *p* = 0.207).
- Similarly, non-fatal stroke, reported in 8 of the 11 studies, was significantly reduced (HR (95% CI): 0.85(0.77–0.95); I² = 0.0%, *p* = 0.696). A sensitivity analysis excluding the ITCA trial yielded consistent results (HR (95%CI): 0.85 (0.77–0.94); I² = 0.0%, *p* = 0.620).
- Hospitalisation for heart failure, reported in 5 of the 11 studies, showed a smaller but statistically significant reduction (HR (95% CI): 0.88(0.77–0.99); I² = 14.6%, *p* = 0.321). A sensitivity analysis excluding the ITCA trial yielded a similar point estimate, although statistical significance was not retained (HR (95%CI): 0.87 (0.75–1.01); I² = 35.1%, *p* = 0.202).

#### Adverse effects included: 

*Severe hypoglycaemia*,* acute pancreatitis and gastrointestinal side effects.*

- Severe hypoglycaemia was reported in 10 of the 11 studies. Across the trials, the absolute risk differences (ARD) between intervention and placebo groups were minimal, generally ranging from − 0.9% to + 0.9%, indicating no consistent increase in risk with GLP-1 receptor agonists.
- Acute pancreatitis was reported in all 11 studies, with ARD values typically below ± 0.3% and overall event rates below 1%, suggesting no meaningful increase in risk compared with placebo.
- In contrast, gastrointestinal side effects were reported in 10 of the 11 studies and were consistently more common in the intervention groups. Across the trials, ARD values ranged from + 0.6% to + 17%. These findings confirm that while the GLP-1 RAs do not materially increase rare adverse events such as pancreatitis and severe hypoglycaemia, gastrointestinal side effects represent a predictable and clinically relevant class effect that often limits tolerability.

### Risk of bias

**Fig. 5:**
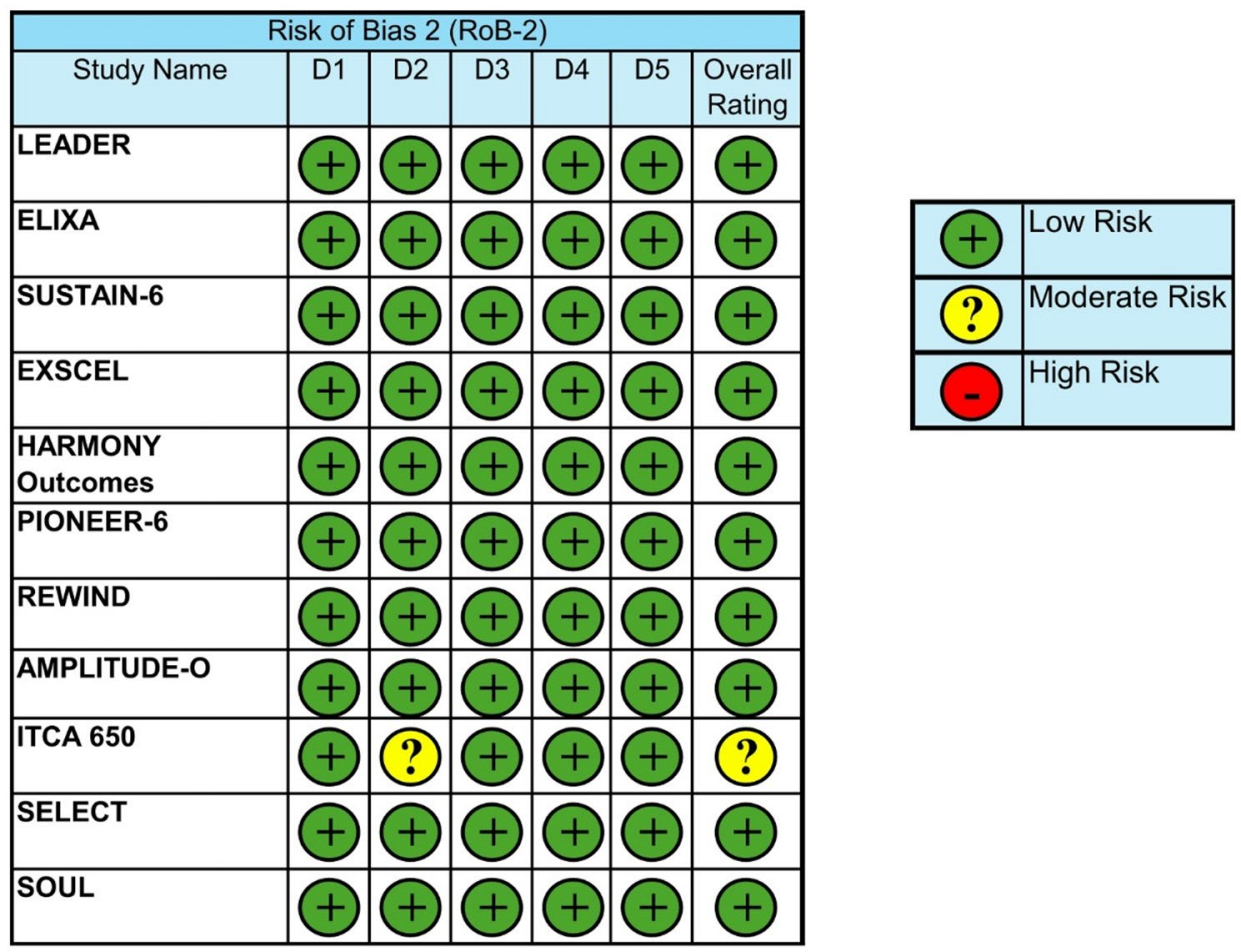
Risk of bias assessment results using Cochrane’s RoB-2 tool for each cardiovascular outcome trial and a key on the right to explain ratings across domains (D1: randomisation process, D2: deviations from the intended interventions, D3: missing outcome data, D4: measurement of the outcome and D5: selection of the reported result) [10]

10 of 11 studies were judged to be at low risk of bias across all RoB2 [10] domains, including randomisation and allocation (D1), deviations from intervention (D2), missing outcome data (D3), measurement of outcome (D4) and selection of reported results (D5). One study (ITCA 650) reported as ‘some concerns’ overall due to risk of bias in D2 due to concerns regarding the implantable device affecting the blinding of the procedure, which may have influenced how the intervention was administered (Fig. 5).

### Certainty of evidence

**Fig. 6:**
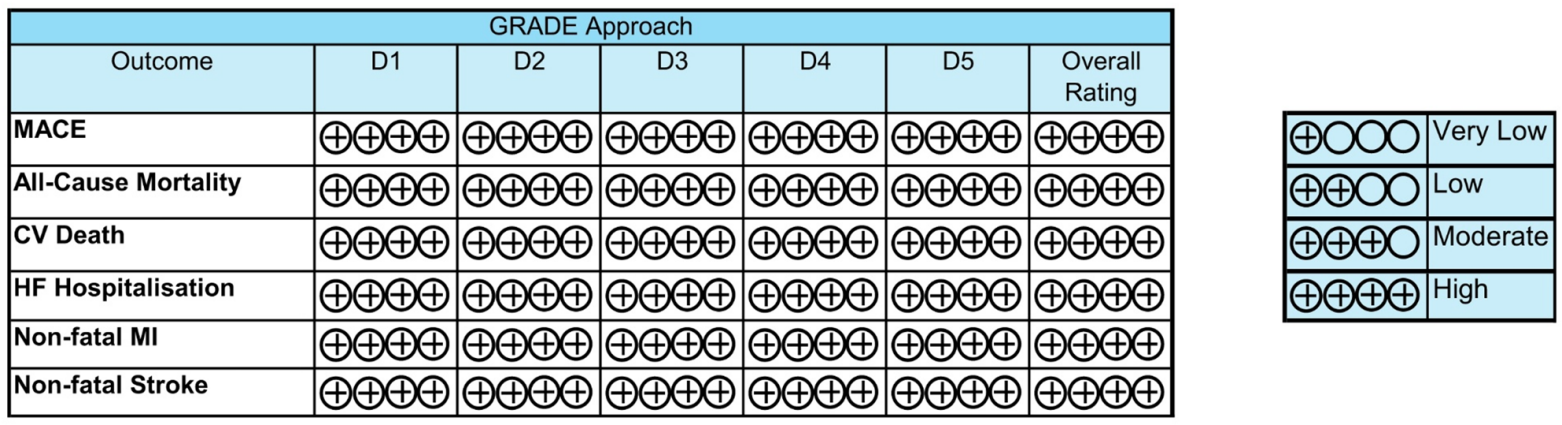
GRADE (Grading of Recommendations, Assessment, Development and Evaluation) approach results to illustrate quality of evidence across outcomes. Key on the right to explain ratings across domains (D1: risk of bias, D2: inconsistency, D3: indirectness, D4: imprecision and D5: publication bias) [11]

All primary and secondary outcomes were judged to have high certainty of evidence across all GRADE approach [11] domains, including risk of bias (D1), inconsistency (D2), indirectness (D3), imprecision (D4), publication bias (D5). As ITCA 650 contributed minimally to the pooled estimate, its potential bias did not materially affect the overall assessment. A post-hoc sensitivity analysis excluding ITCA 650 validated the robustness of the pooled hazard ratio (Supplementary Figure D6). Therefore, D1 was judged as ‘high certainty’ overall for each outcome (Fig. 6).

## Discussion

Despite recent advancements in cardiovascular prevention and treatment, cardiovascular disease remains the leading cause of death worldwide, particularly among high-risk populations such as those with type 2 diabetes and/or hypertension [1]. The persistently elevated risk amongst these trial populations demonstrates the limitations of current management strategies and highlights the need for therapies that address the link between metabolic disease and direct cardiovascular pathology.

The results of this systematic review and meta-analysis demonstrate that glucagon-like peptide-1 receptor agonists (GLP-1RAs) reduce the risk of major adverse cardiovascular events (MACE). The benefits of these drugs likely extend beyond their current use in the treatment of diabetes and obesity to improve glycaemic control, as cardiovascular benefits have been observed across diabetic and pre-diabetic populations. Sensitivity analyses confirmed that the cardioprotective effect of GLP-1RAs is largely independent of glycaemic status, suggesting a direct cardiovascular benefit in patients with diabetes. GLP-1RAs therefore function as a dual-action therapy by improving metabolic risk factors such as type 2 diabetes and hyperglycaemia and directly protecting the cardiovascular system.

Preclinical and translational studies provide plausible explanations for these observed cardiovascular effects. GLP-1 receptors have been identified within myocardial tissue and autonomic centres [21–23], with murine studies demonstrating effects on heart rate, endothelial function and regulation of the autonomic nervous system [24–26]. These effects work alongside indirect benefits, including weight loss, reduced blood pressure, and improved lipid metabolism, which, in turn, may enhance protection against atherosclerotic disease. Together, these direct and indirect effects provide a rationale for the observed reduction of MACE in our meta-analysis.

In the meta-analysis, the different follow-up periods across studies were included, which could increase the chance of heterogeneity. Obtaining the individual data to deal with this is challenging, as this study is based on large sets of data. Nevertheless, the random-effects meta-analyses were applied, and the meta-regression analysis showed that the association between the different follow-up periods and the HR estimates was not significant (*p* > 0.05).

A significant reduction in MACE is confirmed by our meta-analysis, consistent with lower cardiovascular and all-cause mortality with the introduction of GLP-1 RAs. While several studies demonstrated non-significant or borderline effects for myocardial infarction, stroke or cardiovascular death, pooling the data revealed significant reductions in MACE and all-cause mortality. The combined evidence from various trials also demonstrated a consistent benefit across trial designs, drug formulations, and patient populations, supporting the conclusion that the cardiovascular benefit is likely a class effect rather than an isolated finding. Although overall findings support a cardioprotective effect of GLP-1 RAs, the ITCA 650 trial showed directionally higher hazard ratios for several outcomes. However, sensitivity analyses excluding this study did not materially alter pooled estimates, supporting the robustness of the findings; statistical significance for hospitalisation for heart failure was attenuated, likely reflecting reduced precision. Alongside this, a subgroup analysis suggested a more pronounced and consistent effect with semaglutide compared with the overall class effect of GLP-1 RAs and may partly explain the moderate heterogeneity observed in the primary analysis. However, as this analysis was conducted post hoc, the findings should be interpreted with caution and considered hypothesis-generating rather than evidence of superiority between agents.

Several prior meta-analyses have examined the cardiovascular effects of GLP-1RAs and, in some cases, included more studies. However, these analyses often included smaller trials with shorter follow-up, which may increase heterogeneity and reduce reliability regarding long-term cardiovascular and safety outcomes. In contrast, this study applied stricter inclusion criteria, restricting the analysis to large randomised cardiovascular outcome trials with a minimum sample size of 3000 participants and a minimum follow-up of at least 12 months. This approach was used to increase statistical power and provide a more thorough evaluation of long-term cardiovascular efficacy and safety. Our findings are broadly consistent with the existing literature while providing a more focused evaluation of higher-quality, more recent evidence, including trials such as SELECT [8] and SOUL [16].

Safety is a critical factor to consider when evaluating long-term cardiometabolic therapies. Evidence from the cardiovascular outcome trials included in this review consistently demonstrates that GLP-1 RAs do not increase the risk of severe hypoglycaemia or acute pancreatitis. These findings directly address the long-standing safety concerns regarding this drug class. The incidence of severe hypoglycaemia was minimal and generally comparable to placebo, reflecting the glucose-dependent mechanism of GLP-1 RAs and contrasting with the higher risk of hypoglycaemia associated with insulin and sulfonylureas. Likewise, although pancreatitis has been a theoretical concern, trial data has consistently shown that the incidence is very low (< 1%) with no significant increase in risk compared to placebo [4, 14, 20].

Conversely, participants receiving GLP-1 RAs were consistently more likely to report experiencing gastrointestinal side effects. The predominant complaints were nausea, vomiting and diarrhoea; in some trials, the incidence was more than 10% higher compared to placebo [14]. These adverse effects are well-recognised class-specific reactions that may restrict tolerance and adherence in clinical practice. To maximise the cardiovascular benefits of GLP-1 RAs at a population level, strategies can be used to improve tolerability, such as patient counselling, gradual dose escalation and the use of long-acting formulations [27]. Overall, GLP-1 RAs have a favourable safety profile, with benefits that significantly outweigh their manageable side effects.

Recent studies have reported that combining GLP-1 receptor agonist and SGLT2 inhibitor therapy improves cardiovascular and metabolic outcomes compared with the SGLT2 inhibitor monotherapy [28]. These advantages are less consistent when compared with GLP-1 RA monotherapy, suggesting that the predominant effect may be driven by GLP-1 RAs themselves [29]. Our findings are consistent with this, as they indicate an additive effect over SGLT2 inhibition alone, but a limited incremental benefit beyond GLP-1 RAs.

Several limitations should be acknowledged in this meta-analysis. Firstly, the use of aggregated trial-level data limits the ability to conduct thorough subgroup analyses when compared to individual patient data. Additionally, variation in study populations, trial design and baseline cardiovascular risk may have influenced the observed outcomes. Differences in dosing regimens were not formally assessed, meaning a potential dose–response relationship could not be explored. While publication bias cannot be entirely excluded, a comprehensive search strategy across multiple databases was used to minimise this risk. Despite these limitations, the inclusion of large, high-quality randomised controlled trials supports the overall reliability of the findings.

Looking ahead, the accumulating body of evidence supporting the cardiovascular benefits of GLP-1 RAs highlights the need to integrate them more widely into routine clinical practice. These therapies should be regarded as key components of larger cardiovascular risk reduction strategies, especially for individuals with established heart disease or type 2 diabetes, in addition to being used as treatments for glycaemic control. High-risk individuals may receive more long-term cardiovascular protection if GLP-1 RAs are initiated earlier. Additional cardiovascular benefits may be provided by combination therapy with other antidiabetic medications, such as SGLT2 inhibitors. Overcoming barriers such as availability, affordability and clinical awareness could broaden implementation in clinical practice. Incorporating GLP-1 RAs into standard treatment pathways has the potential to substantially lower cardiovascular morbidity and mortality among high-risk populations.

**Table 1:** Data extraction table for cardiovascular outcome trials, covering study design, intervention details, baseline characteristics and outcomes

Basic Info	Study Design					Intervention Details		Baseline Characteristics				Outcomes and Conclusions											
RCT Trial	Sample Size	Intervention (*n*)	Comparator (*n*)	Median Duration (yrs)	Population	Drug	Dose	Age (mean)	Gender (% female)	HbA1c (%)	CVD (%)	Statistical Test Used	Primary Outcomes	Incidents (Primary - Intervention)	Incidents (Primary - Comparator)	Hazard Ratio	Secondary Outcomes	Incidents (Secondary - Intervention)	Incidents (Secondary - Comparator)	Hazard Ratio	Severe Hypoglycaemia (%)	Pancreatitis (%)	GI Side effects (%)
LEADER	9340	4668	4672	3.8	Mean diabetes duration 13 years; T2DM at high CV risk	Liraglutide	1.8 mg or max tolerated dose	64	36	8.7	81	Cox regression model including only treatment group as covariate	Composite CV outcome (MACE): CV death, MI, stroke	608	694	0.87 (95% Cl: 0.78–0.97); *P* < 0.001 noninferiority; *P* = 0.01 superiority	CV death, all-cause death, MI, stroke, HF hosp.	CV death: 219; all cause death: 381	CV death: 278; all cause death: 447	CV death (0.78; 95% CI: 0.66–0.93; *P* = 0.007), all-cause death (0.85; 95% CI: 0.74–0.97; *P* = 0.02)	2.4 (liraglutide); 3.3 (placebo); *P* = 0.02	0.4 (liraglutide); 0.5 (placebo); *P* = 0.44	9.5 (liraglutide), 7.3 (placebo); *P* < 0.001
ELIXA	6068	3034	3034	2.1	T2DM and recent ACS	Lixisenatide	10-20mcg S/C once daily	60	31	7.7	100	Cox proportional-hazards model with study group and geographocal region as covariates	Composite CV outcome (MACE): CV death, MI, stroke	406	399	1.02 (95% Cl: 0.89–1.17); *P* < 0.001 noninferiority; *P* = 0.81 superiority	HF hosp., all-cause death	CV death: 156; MI: 270; stroke: 67; HF hosp.: 456	CV death: 158; MI: 261; stroke: 60; HF hosp.: 469	HF hosp. (HR 0.96; 95% Cl: 0.75–1.23), all-cause death (HR 0.94; 95% Cl: 0.78–1.13)	0.5 (lixisenatide), 0.8 (placebo)	0.2 (lixisenatide), 0.3 (placebo)	4.9 (lixisenatide), 1.2 (placebo); *P* < 0.001
SUSTAIN-6	3297	1648	1649	2.1	T2DM on standard care regimen	Semaglutide S/C	0.5 mg or 1 mg once weekly for 104 wks	65	39	8.7	83	Cox proportional-hazards model with pooled treatment as a fixed factor	Composite CV outcome (MACE): CV death, MI, stroke	108	146	0.74 (95% Cl: 0.58–0.95); *P* < 0.001 noninferiority	Non-fatal MI, non-fatal stroke, CV death	Non-fatal MI: 47; Non-fatal stroke: 27; CV death: 44	Non-fatal MI: 64; Non-fatal stroke: 27; CV death: 46	Nonfatal MI: 0.74 (95% CI: 0.51–1.08; *P* = 0.12), Nonfatal Stroke: 0.61 (95% CI: 0.38–0.99; *P* = 0.04), CV death: 0.98 (95% CI: 0.65–1.48; *P* = 0.92)	22.1 (semaglutide), 21.2 (placebo)	0.5 (semaglutide), 0.7 (placebo)	**52** (semaglutide), 35 (placebo)
EXSCEL	14,752	7356	7396	3.2	T2DM with or without CVD	Exenatide S/C extended release	2 mg once weekly	62	38	8	73	Cox proportional-hazards model	Composite CV outcome (MACE): CV death, MI, stroke	839	905	0.91 (95% Cl: 0.83-1.00); *P* < 0.001 for noninferiority; *P* = 0.06 superiority	All-cause death, CV death, stroke, HF hosp.	All-cause death:507; CV death: 340; MI: 483; Stroke: 187; HF hosp.: 219	All-cause death:584; CV death: 383; MI: 493; Stroke: 218; HF hosp.: 231	Non-fatal MI: 0.91 (95% CI: 0.81–1.02), Non-fatal Stroke: 0.90 (95% CI: 0.75–1.08), HF hosp.: 0.99 (95% CI: 0.79–1.24), ACS: 1.00 (95% CI: 0.81–1.24)	3.4 (exenatide), 3.0 (placebo)	0.4 (exenatide), 0.3 (placebo)	Not recorded
HARMONY Outcomes	9463	4731	4732	1.6	T2DM and CVD	Albiglutide	30-50 mg based on glycaemic response and tolerability	64	30	8.7	100	Cox proportional hazards regression, with treatment group as the only explanatory variable	Composite cardiovascular events (CV death, MI, stroke)	338	428	0.78 (95% CI: 0.68–0.90); *P* < 0.0001 noninferiority; *P* = 0.0006 superiority	CV death, MI, Stroke, All cause death	CV death:122; MI: 181; Stroke: 94; all cause death: 196	CV death:130; MI: 240; Stroke: 108; all cause death: 205	MI: 0.75 (95% CI: 0.61–0.90; *P* = 0.003), stroke: 0.86 (95% CI: 0.66–1.14; *P* = 0.3), CV death: 0.93 (95% CI: 0.73–1.19; *P* = 0.578)	1.0 (albiglutide), 1.0 (placebo	< 0.1 (albiglutide), < 0.1 (placebo)	2.0 (albiglutide), 2.0 (placebo)
PIONEER-6	3183	1591	1592	1.3	≥50 years old with established CV or chronic kidney disease, or ≥60 years old with CV risk factors only	Semaglutide Oral	14 mg max daily dose	66	32	8.2	85	A stratified Cox proportional-hazards model was used for the primary outcome analysis, with trial group as a fixed factor.	Composite cardiovascular events (CV death, MI, stroke)	61	76	0.79 (95% CI: 0.57–1.11); *P* < 0.001 noninferiority	CV death, nonfatal MI, nonfatal stroke, all-cause death	all-cause death: 23; CV death: 15; non-fatal MI: 37; non-fatal stroke: 12; HF hosp.:21	all-cause death: 45; CV death: 30; non-fatal MI: 31; non-fatal stroke: 16; HF hosp.:24	CV death: 0.49 (95% CI: 0.27–0.92); Nonfatal MI: 1.18 (95% CI: 0.73–1.90); Nonfatal stroke: 0.74 (95% CI: 0.35–1.57); all-cause death: 0.51 (95% CI: 0.31–0.84)	1.4 (semaglutide), 0.8 (placebo)	0.1 (semaglutide), 0.2 (placebo)	6.8 (semaglutide), 1.6 (placebo)
REWIND	9901	4949	4952	5.4	T2DM with/ without CVD or risk factors;≥ 50 years old	Dulaglutide	1.5 mg S/C once a week	66	46	7.3	31	Kaplan-Meier estimates used to generate cumulative risks. Cox proportional hazards models used for effect of intervention on outcome and to estimate HRs and 95% CIs.	Composite cardiovascular events (CV death, MI, stroke)	594	663	0.88 (95% CI: 0.79–0.99); *P* = 0.026)	CV death, nonfatal MI, nonfatal stroke	MI: 223; stroke: 158; cvs death: 317; all-cause death: 536; HF: 213	MI: 231; stroke: 205; cvs death: 346; all-cause death: 592; HF: 226	CV death: 0.91 (95% CI: 0.78–1.06; *P* = 0.21); Nonfatal MI: 0.96 (95% CI: 0.79–1.16; *P* = 0.65); Nonfatal stroke: 0.76 (95% CI: 0.61–0.95; *P* = 0.017); all-cause death: 0.90 (95% CI: 0.80–1.01; *P* = 0.067)	1.3 (dulaglutide), 1.5 (placebo)	0.5 (dulaglutide), 0.3 (placebo)	47.4 (dulaglutide), 34.1 (placebo)
AMPLITUDE-O	4076	2717	1359	1.8	T2DM and CVD or renal disease	Efpeglenatide	4 mg or 6 mg	65	33	8.9	76	Kaplan–Meier curves for cumulative risks. Cox proportional-hazards models adjusted for geographic region. Randomisation stratification factor to estimate HR and 95% CI for effect of efpeglenatide (dose groups combined) on outcomes.	Composite cardiovascular events (CV death, MI, stroke)	189	125	0.73 (95% CI: 0.58–0.92); *P* < 0.001 noninferiority; *P* = 0.007 superiority [double checked and this is correct]	Non-fatal MI, non-fatal stroke, HF, CV death, all-cause death	MI: 91; Stroke: 47; CV death: 75; all-cause mortality: 111; HF: 40	MI: 58; Stroke: 31; CV death: 50; all-cause mortality: 69; HF: 31	Non-fatal MI: 0.78 (95% CI: 0.55–1.10), non-fatal stroke: 0.80 (95% CI: 0.48–1.31), HF: 0.61 (95% CI: 0.38–0.98), CV death: 0.72 (95% CI: 0.50–1.03), all-cause death: 0.78 (95% CI: 0.58–1.06)	0.9 (efpeglenatide), 1.0 (placebo)	0.4 (efpeglenatide), 0.5 (placebo)	3.3 (efpeglenatide), 1.8 (pacebo)
ITCA 650	4156	2075	2081	1.8	T2DM and either a history of CVD or current kidney disease (eGFR: 25.0–59.9 ml/ min/ 1.73 m2 body-surface area) plus ≥ 1 CV risk factor	Exenatide Osmotic mini pump Continuous SC	60mcg per day	63	37.5 (ITCA); 35.9 placebo	8	76	HRs, 95% CIs and P values for time-to-event analyses were derived from a stratified Cox proportional hazards model.	First MACE (nonfatal MI, nonfatal stroke, or death from CV or undetermined causes)	95	79	1.21 (95% CI: 0.90–1.63); *P* = 0.004 non-inferiority	All-cause mortality, cvs death, non-fatal MI, non-fatal stroke; HF hosp.	all-cause mortality: 49; cvs death: 28; non-fatal MI: 37; non-fatal stroke: 23; HF hosp. 16	all-cause mortality: 41; cvs death: 23; non-fatal MI: 28; non-fatal stroke: 23; HF hosp. 17	CV death:1.22 (95% CI: 0.70–2.12); non-fatal MI: 1.33 (95% CI: 0.82–2.17); non-fatal stroke: 1.00 (95% CI: 0.56–1.79); all-cause mortality: 1.20 (95% CI: 0.79–1.81); HF hosp.: 0.95 (95% CI: 0.48–1.88)	0.4 (ITCA 650), 0.2 (placebo)	0.4 (ITCA 650), 0.1 (placebo)	Nausea − 20.6 (ITCA 650), 3.6 (placebo); Vomiting − 13.5 (ITCA 650), 1.2 (placebo); diarrhoea − 7.8 (ITCA 650), 3.5 (placebo)
SELECT	17,604	8803	8801	3.3	≥ 45 year old; pre-existing CVD and BMI ≥ 27; no history of diabetes	Semaglutide S/C	2.4 mg once weekly	62	28	5.8	> 75 previous MI; ~25 chronic HF	Cause-specific HRs and 95% CI using Cox proportional hazards model with randomised assignment (semaglutide or placebo) as fixed factor. One-sided P values from score test.	Composite cardiovascular events (CV death, MI, stroke)	569	701	0.80 (95% CI: 0.72–0.90); *P* < 0.001 noninferiority; *P* = 0.046 superiority	CV death, HF, all- cause death, non-fatal MI, non-fatal stroke, HF hosp.	cvs death: 223; HF: 300; all-cause death: 375; non-fatal MI: 234; non-fatal stroke: 154; HF hosp.: 109	cvs death: 262; HF: 361; all-cause death: 458; non-fatal MI: 322; non-fatal stroke: 165; HF hosp.: 122	CV death: 0.85 (95% CI: 0.71–1.01; *P* = 0.07), HF: 0.82 (95% CI: 0.71–0.96), all-cause death: 0.81 (95% CI: 0.71–0.93)	Not recorded	0.2 (semaglutide), 0.3 (placebo)	10.0 (semaglutide), 2.0 (placebo)
SOUL	9650	4825	4825	4.1	≥ 50 years old, had T2DM with a glycated Hb level 6.5–10.0%, atherosclerotic CV disease, CKD, or both	Semaglutide Oral	Once daily 14 mg max dose	66	28.90	8	57	Cox proportional-hazards model with randomized group assignment as a fixed factor	MACE (death from CV causes, nonfatal MI, or nonfatal stroke), assessed in a time-to-first-event analysis	579	668	0.86; 95% confidence interval, 0.77 to 0.96; *P* = 0.006	CV death, MI, Stroke, All cause death, HF	CV death: 301; MI: 200; stroke: 164; all-cause death: 528; HF: 146	CV death: 320; MI: 268; stroke: 171; all-cause death: 577; HF: 167	CV death: 0.93 (95% CI: 0.80–1.09), MI: 0.73 (95% CI: 0.61–0.88), stroke: 0.95 (95% CI: 0.76–1.17), all-cause death: 0.91 (95% CI: 0.80–1.02)	1.6 (semaglutide), 1.7 (placebo)	0.4 (semaglutide), 0.4 (placebo)	5.0 (semaglutide), 4.4 (placebo)

## Supplementary Information

Below is the link to the electronic supplementary material.

Supplementary Material 1

Supplementary Material 2

Supplementary Material 3

Supplementary Material 4

## Data Availability

All data is available in the manuscript and associated supplementary files.
