# Supplementary material for: The long-term cardiovascular safety and efficacy of glucagon-like peptide-1 (GLP-1) receptor agonists in high-risk cardiovascular populations: a systematic review and meta-analysis
Source: Cardiovasc Diabetol Endocrinol Rep. 2026 May 1;12:36. doi: 10.1186/s40842-026-00295-3 (PMC13134098; doi:10.1186/s40842-026-00295-3)
Supplement: Supplementary file 1 — Supplementary Material 1 [file 40842_2026_295_MOESM1_ESM.docx]

# Project Information

| Review Title | Systematic review and meta-analysis of the long-term cardiovascular safety and efficacy of glucagon-like peptide-1 (GLP-1) receptor agonists in high-risk cardiovascular populations |
| --- | --- |
| Project Lead | Dr Simon Cork |
| Team Members | Kezia Peter, Ocin Roka, Emma Sepp, Maya Warburton, Dr Jufen Zhang |
| Date | 12/05/2025 |
| Institution(s) | Anglia Ruskin University, School of Medicine |

# Background

Describe the population and problem or phenomenon of interest and contextualize it.

| Cardiovascular disease (CVD) is the leading cause of morbidity and mortality worldwide and the risk especially heightens for those with a background of Type 2 Diabetes and other metabolic disorders. This population at higher risk of CVD are disproportionately impacted by atherogenic risk factors such as dyslipidaemia, hypertension, chronic inflammation, and endothelial dysfunction. To improve long-term outcomes in high-risk groups, there is an urgent requirement for effective therapeutic interventions for CVD.  Over the past few years, glucagon-like peptide-1 receptor agonists (GLP-1 RAs) have surfaced as potent glucose-lowering agents. Simply beyond optimising glycaemic control, this drug class may have capacity to reduce cardiovascular risk too. The underlying mechanism of GLP-1 RAs is to enhance insulin secretion by mimicking incretin to enhance glucose-dependent insulin secretion, suppress glucagon release, delay gastric emptying and reduce appetite. Recently, GLP-1 RAs have demonstrated various pleiotropic effects including weight loss, blood pressure reduction and improved lipid profiles.  There have been numerous large-scale cardiovascular outcome trials (CVOTs) evaluating the safety and efficacy of GLP-1 RAs in patients at high cardiovascular risk, with hopeful indications that demonstrate reductions in major adverse cardiovascular events (MACE). Regardless of these encouraging results, the long-term impact of GLP-1 RAs in cardiovascular health is unclear in patients with established CVD or multiple cardiovascular risks. There are a range of outcomes reported by these trials due to the variability of trial design, patient characteristics and follow-up duration. As a result, this necessitates a comprehensive synthesis of the current data on the long-term cardiovascular safety and efficacy of GLP-1 receptor agonists in high-risk populations to inform clinical decision-making. |
| --- |

# Objective

Describe the justification for this review and why it/the information it collects is important.

| **Primary objective:**   - To evaluate the long-term cardiovascular safety and efficacy of glucagon-like peptide-1 (GLP-1) receptor agonists in adults at high risk of cardiovascular disease.   **Specific review questions:**   - Do GLP-1 receptor agonists reduce the incidence of major adverse cardiovascular events (MACE) compared to placebo or standard care in high-risk cardiovascular populations? - What is the effect of GLP-1 receptor agonists on individual cardiovascular outcomes such as cardiovascular mortality, non-fatal myocardial infarction, and stroke? - Are GLP-1 receptor agonists associated with increased adverse effects, such as severe hypoglycaemia, pancreatitis and gastrointestinal side effects? |
| --- |

# Search Strategy

| Databases |
| --- |
| List the bibliographic databases to be searched. |
| PubMed, Embase via OVID, the Cochrane Library |
| Hand Searching |
| List journals or websites that will be hand searched for relevant articles. |
|  |
| Experts or Stakeholders |
| If experts are being contacted for additional grey literature or research, list them. |
|  |
| Reference Searches |
| If forward or backward citations will be performed, detail them here. |
|  |

# Eligibility Criteria

Operationalize your PICO (or other framework) concepts by explicitly stating what would and would not meet inclusion.

| PICOS | Inclusion Criteria | Exclusion Criteria |
| --- | --- | --- |
| Population | - Adults (≥18 years) only at high risk for cardiovascular disease (CVD) or with established CVD (coronary artery disease, heart failure, prior myocardial infarction, stroke). - Patients with comorbid conditions associated with cardiovascular risk - type 2 diabetes, hypertension, dyslipidaemia. | - Studies involving paediatric or adolescent populations (<18 years). - Patients without high cardiovascular risk or established CVD. |
| Intervention | - Studies evaluating GLP-1 receptor agonists (Liraglutide, Lixisenatide, Semaglutide, Exenatide, Albiglutide, Dulaglutide, Efpeglenatide). | - Studies evaluating drugs other than GLP-1 receptor agonists or using combinations that confound the assessment of GLP-1 agonist efficacy/safety. |
| Comparison | - Placebo | - Other drugs such as SGLT-2, insulin and metformin |
| Outcomes | - Primary: Cardiovascular outcomes - major adverse cardiovascular events [MACE] - Secondary: cardiovascular mortality, non-fatal myocardial infarction, non-fatal stroke, heart failure - Adverse Effects: Safety outcomes (severe hypoglycaemia, pancreatitis, GI side effects). | - Renal, diabetic or weight outcomes - Studies without cardiovascular data |
| Study Design | - Randomised controlled trials (RCTs) - Minimum total sample size of 3000 participants - Minimum follow-up period of 12 months to evaluate long-term outcomes. - Peer-reviewed articles, published in English. - Studies published within the last 10 years to reflect current clinical practices and drug development. | - Case reports, case series, cross-sectional studies, or editorials, post-hoc analyses. - Grey literature - Studies with a follow-up period shorter than 12 months. - Publications in a different language other than English - Studies published more than 10 years ago. |

# Data Extraction

Provide a description of methods used to collect data from included studies (e.g. categories of data you intend to collect, how many people will conduct extraction, etc.).

| Data extraction is independently conducted by two reviewers using the PRISMA guidelines. Extracted data will include study characteristics (author, year, study design), population details (sample size, age, sex, cardiovascular risk profile), intervention details (type, duration of treatment), comparator, primary outcomes (Major adverse cardiovascular event), secondary outcomes (cardiovascular mortality, non-fatal myocardial infarction, non-fatal stroke, hospitalisation for heart failure), adverse effects: safety outcomes (severe hypoglycaemia, pancreatitis, GI side effects), follow-up duration, etc. Discrepancies between reviewers will be resolved through discussion or consultation with a third reviewer. |
| --- |

# Study Quality Assessment

If applicable, describe the tool(s) you will use to assess risk of bias.

| The quality of each included study and outcomes will be independently assessed by two reviewers. Discrepancies will be resolved through discussion and referral to a third reviewer as necessary. The Cochrane Risk of Bias 2 (RoB 2) tool will be used to evaluate bias across five domains: randomisation process, deviations from the intended interventions, missing outcome data, measurement of the outcome and selection of the reported result. Each study will be assigned a risk of bias rating of ‘low risk’, ‘some concerns’ or ‘high risk’ in each domain and an overall rating.  The overall rating for risk of bias is to be obtained through the RoB 2 tool, included as part of the Grading of Recommendations, Assessment, Development and Evaluation (GRADE) approach. The GRADE approach will assess the quality of the evidence for each outcome across five domains: risk of bias, inconsistency, indirectness, imprecision and publication bias. Each outcome will be rated as very low, low, moderate and high certainty. Summary of findings tables will be constructed to present the quality of evidence across the included outcomes and studies. |
| --- |

# Data Synthesis

Describe how you will analyse and summarise the included study results.

| We will perform a narrative synthesis of studies to include the type of intervention, study population characteristics, reported primary and secondary outcomes and adverse effects. Statistical analysis will be conducted using Stata version 18 statistical software. Hazard Ratio (HR) with 95% CI will be used to assess the outcomes between the two treatment groups. Heterogeneity will be assessed using chi-squared tests as well as I-squared test. Forest plots will be used to represent the results generated from the random-effects meta-analysis graphically. The pooled HR and the degree of heterogeneity will also be presented. Publication bias will be minimised by comprehensive literature searching. |
| --- |

# Project Tools

List the tools and software programs you plan to use in the course of the review.

| To streamline the review process, Covidence, an automation tool for systematic reviews, will be used to manage reference screening and selection All retrieved citations will be imported into Covidence, where duplicates will be automatically removed. Additional duplicates will be identified and removed manually using RefWorks. |
| --- |

# Research Team Member Roles

Describe the different tasks on the review and who will be responsible for what.

| Task | Description | Team Member Responsible |
| --- | --- | --- |
| Data analysis, Manuscript preparation | Provide an objective assessment of the protocol to ensure it is scientifically sound, ethically appropriate, and compliant with relevant guidelines. Review the study design, methodology, risk-benefit analysis, and ethical considerations such as informed consent and confidentiality. | Kezia Peter |
| Data analysis, Manuscript preparation | Provide an objective assessment of the protocol to ensure it is scientifically sound, ethically appropriate, and compliant with relevant guidelines. Review the study design, methodology, risk-benefit analysis, and ethical considerations such as informed consent and confidentiality. | Ocin Roka |
| Manuscript preparation | Draft, edit, and finalise the discussion section of the systematic review, ensuring it accurately interprets the results, discusses limitations, and places findings in the context of existing evidence. | Emma Sepp |
| Manuscript preparation | Draft, edit, and finalise the discussion section of the systematic review, ensuring it accurately interprets the results, discusses limitations, and places findings in the context of existing evidence. | Maya Warburton |
| Supervisor | Provide overall guidance and oversight for the systematic review process, including methodological advice, feedback on protocol development, data synthesis, and final write-up. | Dr Simon Cork |
| Statistician, Manuscript preparation | Advise on statistical methods for data synthesis, conduct or verify statistical analyses (e.g., meta-analysis), assess heterogeneity and publication bias, and assist in interpreting the statistical results. | Dr Jufen Zhang |

# References

| This Review Protocol was originally created by Sarah Visintini, Maritime SPOR SUPPORT Unit and adapted from the following resources:  Cochrane Public Health Group. (2011) Guide for developing a Cochrane protocol. Retrieved from: http://ph.cochrane.org/sites/ph.cochrane.org/files/uploads/Guide%20for%20PH%20protocol_Nov%202011_final%20for%20website.pdf.  Dartmouth Biomedical Libraries. (2012). Systematic Review Steps. Retrieved from http://www.dartmouth.edu/~library/biomed/services/lgr/docs/SR-Steps-Roles-revised.docx  Durham University Community. (2009). Template for a Systematic Literature Review Protocol. Retrieved from https://community.dur.ac.uk/ebse/resources/templates/SLRTemplate.pdf.  Warwick Medical School. (n.d.) Protocol Template: Systematic Review. Retrieved from http://www2.warwick.ac.uk/fac/med/staff/bridle/sr/protocol_template.doc.  World Health Organization. (2011). Review Protocol Template. Retrieved from http://www.who.int/hrh/education/Rec1_CPDforfacultyteachingstaff.pdf. |
| --- |
