# Supplementary material for: The long-term cardiovascular safety and efficacy of glucagon-like peptide-1 (GLP-1) receptor agonists in high-risk cardiovascular populations: a systematic review and meta-analysis
Source: Cardiovasc Diabetol Endocrinol Rep. 2026 May 1;12:36. doi: 10.1186/s40842-026-00295-3 (PMC13134098; doi:10.1186/s40842-026-00295-3)
Supplement: Supplementary file 2 — Supplementary Material 2 [file 40842_2026_295_MOESM2_ESM.docx]

**Databases Searched:**

- CLIB – The Cochrane Library
- Embase – Embase via Ovid
- PubMed

**Search Terms:**

- *("Humans")*
- *AND ("Heart Disease Risk Factors")*
- *AND ("Cardiovascular Diseases")*
- *AND ("Glucagon-Like Peptide-1 Receptor Agonists"*
- *OR "Glucagon-Like Peptide 1"*
- *OR "Glucagon-Like Peptide-1 Receptor"*
- *OR "Exenatide"*
- *OR "Semaglutide"*
- *OR "Lixisenatide"*
- *OR "Efpeglenatide"*
- *OR "Dulaglutide"*
- *OR "Liraglutide")*
- *AND ("Hypoglycemia"*
- *OR "Incidence"*
- *OR "Heart Failure"*
- *OR "Hospitalisation"*
- *OR "Myocardial Infarction"*
- *OR "Pancreatitis"*
- *OR "Stroke")*
- *AND ("Randomised Controlled Trial")*

**Grey Literature:**

- None used, this was excluded.

**Inclusion Criteria:**

| Population | - Adults (≥18 years) only at high risk for cardiovascular disease (CVD) or with established CVD (coronary artery disease, heart failure, prior myocardial infarction, stroke). - Patients with comorbid conditions associated with cardiovascular risk - type 2 diabetes, hypertension, dyslipidaemia. |
| --- | --- |
| Intervention | - Studies evaluating GLP-1 receptor agonists (Liraglutide, Lixisenatide, Semaglutide, Exenatide, Albiglutide, Dulaglutide, Efpeglenatide). |
| Comparison | - Placebo |
| Outcomes | - Primary: Cardiovascular outcomes - major adverse cardiovascular events [MACE] - Secondary: cardiovascular mortality, non-fatal myocardial infarction, non-fatal stroke, heart failure - Adverse Effects: Safety outcomes (severe hypoglycaemia, pancreatitis, GI side effects). |
| Study Design | - Randomised controlled trials (RCTs) - Minimum total sample size of 3000 participants. - Minimum follow-up period of 12 months to evaluate long-term outcomes. - Peer-reviewed articles, published in English. - Studies published within the last 10 years to reflect current clinical practices and drug development. |

**Exclusion Criteria:**

| Population | - Studies involving paediatric or adolescent populations (<18 years). - Patients without high cardiovascular risk or established CVD. |
| --- | --- |
| Intervention | - Studies evaluating drugs other than GLP-1 receptor agonists or using combinations that confound the assessment of GLP-1 agonist efficacy/safety. |
| Comparison | - Other drugs such as SGLT-2, insulin and metformin |
| Outcomes | - Renal, diabetic or weight outcomes - Studies without cardiovascular data |
| Study Design | - Case reports, case series, cross-sectional studies, or editorials, post-hoc analyses. - Grey literature - Studies with a follow-up period shorter than 12 months. - Publications in a different language other than English - Studies published more than 10 years ago. |
