# Supplementary material for: The long-term cardiovascular safety and efficacy of glucagon-like peptide-1 (GLP-1) receptor agonists in high-risk cardiovascular populations: a systematic review and meta-analysis
Source: Cardiovasc Diabetol Endocrinol Rep. 2026 May 1;12:36. doi: 10.1186/s40842-026-00295-3 (PMC13134098; doi:10.1186/s40842-026-00295-3)
Supplement: Supplementary file 4 — Supplementary Material 4 [file 40842_2026_295_MOESM4_ESM.docx]

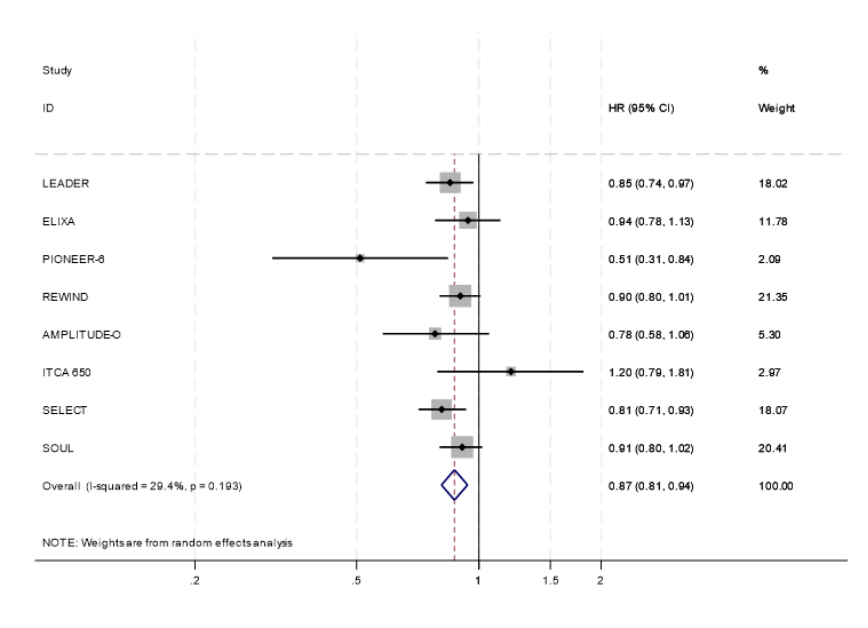


**Figure 1: Forest plot diagram between GLP-1 receptor agonists and placebo for all-cause mortality according to random effects analysis.**


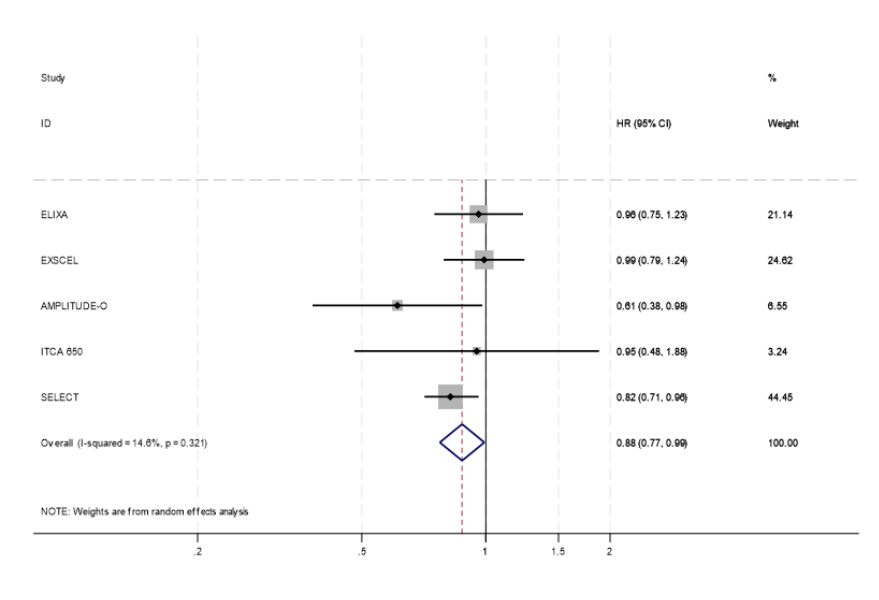


**Figure 2: Forest plot diagram between GLP-1 receptor agonists and placebo for heart failure hospitalisation according to random effects analysis.**


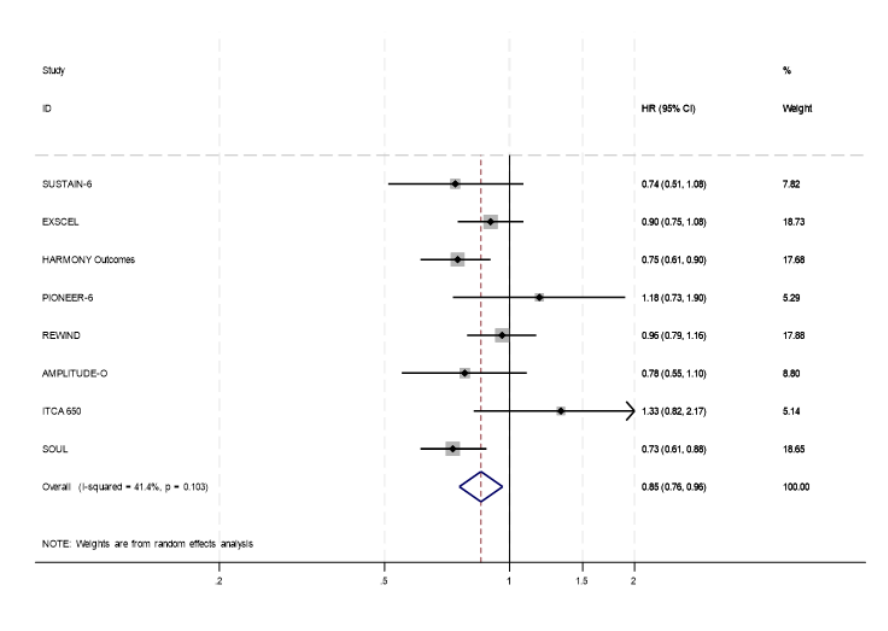


**Figure 3: Forest plot diagram between GLP-1 receptor agonists and placebo for non-fatal myocardial infarction according to random effects analysis.**
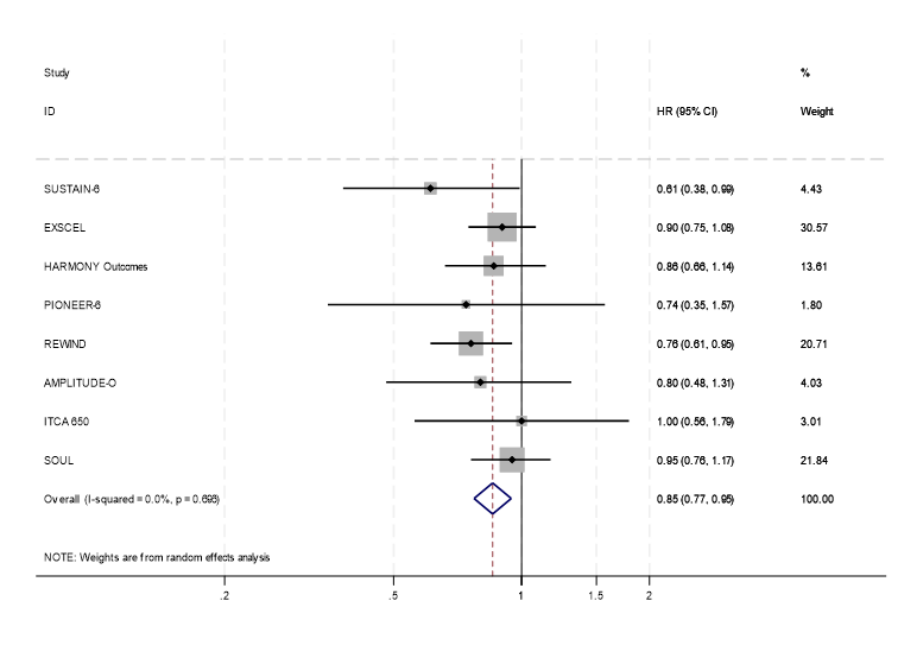
  **Figure 4: Forest plot diagram between GLP-1 receptor agonists and placebo for non-fatal stroke according to random effects analysis.**


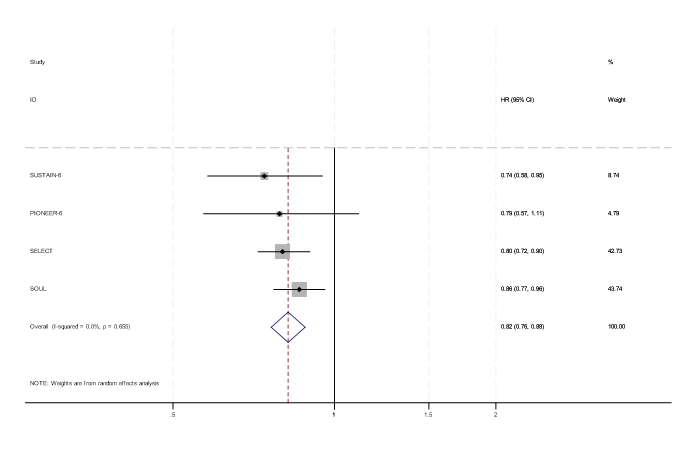


**Figure 5: Forest plot diagram comparing MACE events across the four trials investigating semaglutide only as a subgroup analysis according to random effects analysis.**


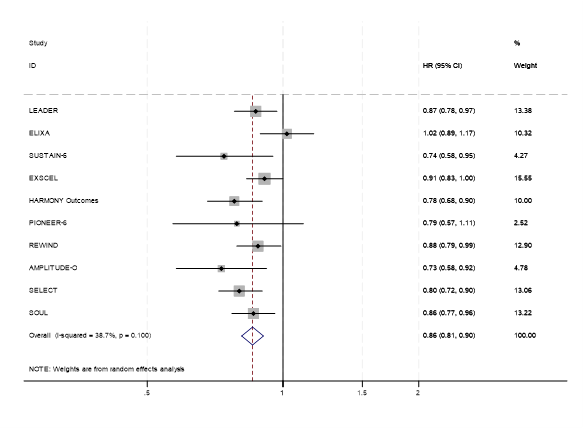


**Figure 6: Forest plot diagram comparing MACE events across all trials excluding ITCA 650 according to random effects analysis.**


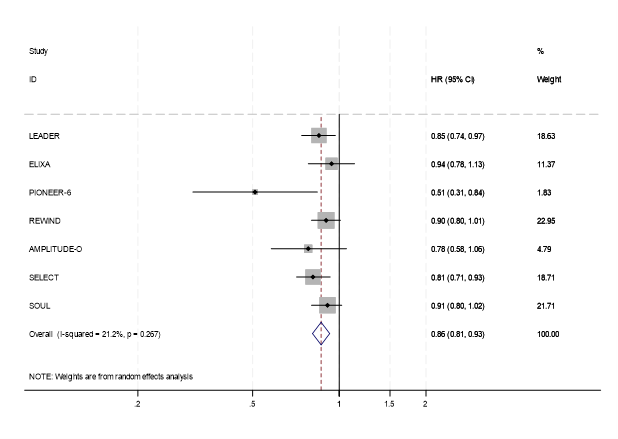


**Figure 7: Forest plot diagram comparing all-cause mortality events across all trials excluding ITCA 650 according to random effects analysis.**


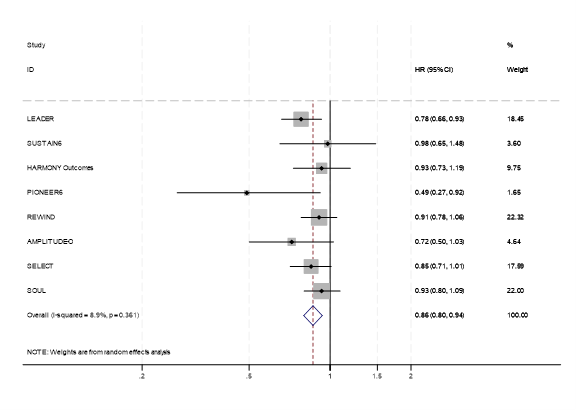


**Figure 8: Forest plot diagram comparing cardiovascular mortality events across all trials excluding ITCA 650 according to random effects analysis.**


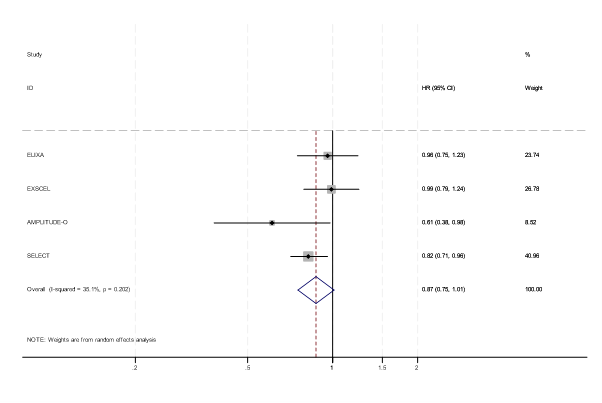


**Figure 9: Forest plot diagram comparing hospitalisation due to heart failure events across all trials excluding ITCA 650 according to random effects analysis.**


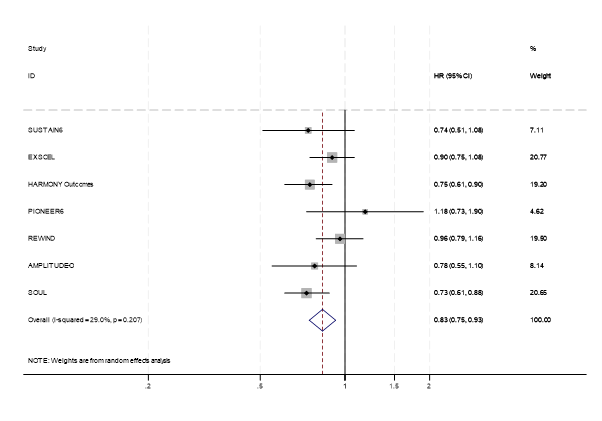


**Figure 10: Forest plot diagram comparing non-fatal myocardial infarction events across all trials excluding ITCA 650 according to random effects analysis.**


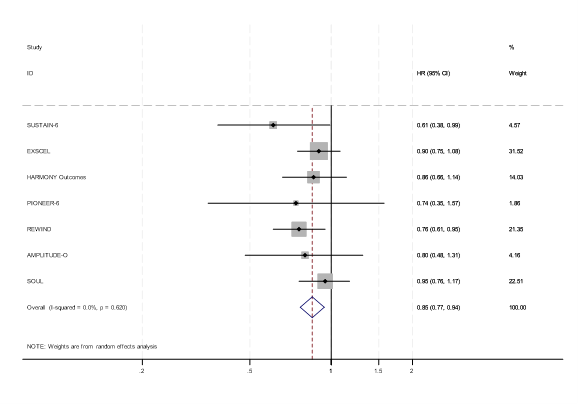


**Figure 11: Forest plot diagram comparing non-fatal stroke events across all trials excluding ITCA 650 according to random effects analysis.**
