# Supplementary material for: The long-term cardiovascular safety and efficacy of glucagon-like peptide-1 (GLP-1) receptor agonists in high-risk cardiovascular populations: a systematic review and meta-analysis
Source: Cardiovasc Diabetol Endocrinol Rep. 2026 May 1;12:36. doi: 10.1186/s40842-026-00295-3 (PMC13134098; doi:10.1186/s40842-026-00295-3)
Supplement: Supplementary file 4 [file 40842_2026_295_MOESM3_ESM.docx]

Outcome	Risk of Bias - ROB-2							GRADE Approach					
	Study Name	Randomisation Process	Deviations from intended interventions	Missing Outcome Data	Measurement of outcome	Selection of reported result	Overall rating	Overall Outcome ROB-2	Inconsistency	Indirectness	Imprecision	Publication Bias	Overall GRADE
MACE	LEADER	Low	Low	Low	Low	Low	Low	High	Not Serious	Not Serious	Not Serious	Undetected	High
	ELIXA	Low	Low	Low	Low	Low	Low						
	SUSTAIN-6	Low	Low	Low	Low	Low	Low						
	EXSCEL	Low	Low	Low	Low	Low	Low						
	HARMONY Outcomes	Low	Low	Low	Low	Low	Low						
	PIONEER-6	Low	Low	Low	Low	Low	Low						
	REWIND	Low	Low	Low	Low	Low	Low						
	AMPLITUDE-O	Low	Low	Low	Low	Low	Low						
	ITCA 650	Low	Some concerns	Low	Low	Low	Some concerns						
	SELECT	Low	Low	Low	Low	Low	Low						
	SOUL	Low	Low	Low	Low	Low	Low						
All-cause mortality	LEADER	Low	Low	Low	Low	Low	Low	High	Not Serious	Not Serious	Not Serious	Undetected	High
	ELIXA	Low	Low	Low	Low	Low	Low						
	SUSTAIN-7	Low	Low	Low	Low	Low	Low						
	EXSCEL	Low	Low	Low	Low	Low	Low						
	HARMONY Outcomes	Low	Low	Low	Low	Low	Low						
	PIONEER-7	Low	Low	Low	Low	Low	Low						
	REWIND	Low	Low	Low	Low	Low	Low						
	AMPLITUDE-O	Low	Low	Low	Low	Low	Low						
	ITCA 651	Low	Some concerns	Low	Low	Low	Some concerns						
	SELECT	Low	Low	Low	Low	Low	Low						
	SOUL	Low	Low	Low	Low	Low	Low						
CV death	LEADER	Low	Low	Low	Low	Low	Low	High	Not Serious	Not Serious	Not Serious	Undetected	High
	ELIXA	Low	Low	Low	Low	Low	Low						
	SUSTAIN-8	Low	Low	Low	Low	Low	Low						
	EXSCEL	Low	Low	Low	Low	Low	Low						
	HARMONY Outcomes	Low	Low	Low	Low	Low	Low						
	PIONEER-8	Low	Low	Low	Low	Low	Low						
	REWIND	Low	Low	Low	Low	Low	Low						
	AMPLITUDE-O	Low	Low	Low	Low	Low	Low						
	ITCA 652	Low	Some concerns	Low	Low	Low	Some concerns						
	SELECT	Low	Low	Low	Low	Low	Low						
	SOUL	Low	Low	Low	Low	Low	Low						
HF Hospitalisation	LEADER	Low	Low	Low	Low	Low	Low	High	Not Serious	Not Serious	Not Serious	Undetected	High
	ELIXA	Low	Low	Low	Low	Low	Low						
	SUSTAIN-9	Low	Low	Low	Low	Low	Low						
	EXSCEL	Low	Low	Low	Low	Low	Low						
	HARMONY Outcomes	Low	Low	Low	Low	Low	Low						
	PIONEER-9	Low	Low	Low	Low	Low	Low						
	REWIND	Low	Low	Low	Low	Low	Low						
	AMPLITUDE-O	Low	Low	Low	Low	Low	Low						
	ITCA 653	Low	Some concerns	Low	Low	Low	Some concerns						
	SELECT	Low	Low	Low	Low	Low	Low						
	SOUL	Low	Low	Low	Low	Low	Low						
Non-fatal MI	LEADER	Low	Low	Low	Low	Low	Low	High	Not Serious	Not Serious	Not Serious	Undetected	High
	ELIXA	Low	Low	Low	Low	Low	Low						
	SUSTAIN-10	Low	Low	Low	Low	Low	Low						
	EXSCEL	Low	Low	Low	Low	Low	Low						
	HARMONY Outcomes	Low	Low	Low	Low	Low	Low						
	PIONEER-10	Low	Low	Low	Low	Low	Low						
	REWIND	Low	Low	Low	Low	Low	Low						
	AMPLITUDE-O	Low	Low	Low	Low	Low	Low						
	ITCA 654	Low	Some concerns	Low	Low	Low	Some concerns						
	SELECT	Low	Low	Low	Low	Low	Low						
	SOUL	Low	Low	Low	Low	Low	Low						
Non-fatal Stroke	LEADER	Low	Low	Low	Low	Low	Low						

Figure 1: Summary of findings table using Cochrane RoB2 (Risk of Bias) tool (Sterne et al, 2019), as part of the GRADE (Grading of Recommendations, Assessment, Development and Evaluation) approach (Schünemann et al, 2013). Final GRADE rating assesses overall quality of evidence.
